# Factors Influencing Outcomes and Survival in Anal Cancer

**DOI:** 10.3390/curroncol31090381

**Published:** 2024-09-02

**Authors:** Hugo C. Temperley, Benjamin M. Mac Curtain, Niall J. O’Sullivan, Cormac Mulhall, Tatiana S. Temperley, Brian J. Mehigan, John O. Larkin, Paul H. McCormick, Colm Kerr, David Gallagher, Colm Bergin, Charles Gillham, Michael E. Kelly

**Affiliations:** 1 Department of Radiology, St. James’s Hospital, D08 NHY1 Dublin, Ireland; 2 Department of Surgery, St. James’s Hospital, D08 NHY1 Dublin, Ireland; 3 Trinity St. James’s Cancer Institute, D08 NHY1 Dublin, Ireland; 4 Department of Infectious Diseases, St. James’s Hospital, D08 NHY1 Dublin, Ireland; 5 Department of Medical Oncology, St. James’s Hospital, D08 NHY1 Dublin, Ireland; 6 Department of Genetics, St. James’s Hospital, D08 NHY1 Dublin, Ireland; 7 Department of Radiation Oncology, St. James’s Hospital, D08 NHY1 Dublin, Ireland

**Keywords:** anal cancer, oncological outcomes, survival, recurrence, treatment response, salvage surgery

## Abstract

Background: We aim to ascertain prognostic factors in the current management of anal cancer within this study. Methods: We reviewed the management and outcomes of anal cancer cases over a seven-year period, inclusive (2016–2023). The primary objectives were to assess the demographic characteristics, clinical presentation, and outcomes of all anal cancer patients within our institution. Kaplan–Meier survival analysis was used to estimate survival differences between cohorts, with statistical significance determined using log-rank testing. Cox proportional hazards regression was utilised to identify prognostic factors. Cox regression hazard ratios were reported along with confidence intervals and *p*-values. Results: The median follow-up time for the study was 29.8 months. Seventy-five patients with anal cancer were included in this study, with 88% (66/75) being squamous cell carcinoma (SCC) and the majority having regional disease (82.7% (62/75)). The median age at diagnosis was 63.4 years (36–94). There was a female preponderance (57.3% (43/75)). In total, 84% (63/75) underwent definitive chemoradiation (*dCRT*), with 7/63 (11.1%) requiring a salvage abdomino-perineal resection (APR) for residual or recurrent disease. Adverse prognostic indicators include those with T4 disease hazard ratio = 3.81, (95% CI 1.13–12.83, * *p* = 0.04), poorly differentiated tumour disease HR = 3.37, (95% CI 1.13–10.02, * *p* = 0.04), having N2 nodal status HR = 5.03, (95% CI 1.11–22.8, * *p* = 0.04), and having metastatic disease at diagnosis HR = 5.8, (95% CI 1.28–26.42, * *p* = 0.02). Conclusion: Presenting characteristics including stage, nodal, and differentiation status remain key prognostic indicators in those diagnosed with anal malignancy.

## 1. Introduction

Anal cancer remains a rare disease, accounting for only about 2% of all gastrointestinal tract (GIT) malignancies [1,2]. Despite this, its diagnosis carries significant clinical, psychological, and public health implications. Anal cancer is largely classified into two subtypes: squamous cell carcinoma (SCC) and adenocarcinoma [1,3]. SCC accounts for the majority (>80%) of anal cancers, with adenocarcinoma (~10–20%), anal melanoma, and basal cell carcinoma occurring less frequently (<3%) [4]. Both SCC and adenocarcinoma share common risk factors, such as human immunodeficiency virus (HIV) infection, history of receptive anal intercourse, and immunosuppression [5,6,7].

There are many challenges in diagnosing anal cancer. Patients may initially present with symptoms such as discomfort, bleeding, or a lump in the anal area, all of which overlap with common, benign conditions [8,9]. Additionally, a large proportion of these neoplasms occur in socio-economically disadvantaged populations, with delays in seeking medical attention compounding issues [9,10,11].

Treatment strategies for anal cancer have evolved slowly over time, especially when compared with colon or rectal neoplasms [12,13]. There is a well-established emphasis on organ preservation in contrast with other GIT malignancies where surgery is the first-line treatment [10,14,15]. This has been the standard of care for anal cancer (anal SCC) since the Nigro protocol of the 1970’s with the first-line option for locoregional anal cancer being chemotherapy with mitomycin-C and 5-flourouracil concurrent with radiotherapy, despite associated toxicities [16,17,18,19,20].

The incidence of anal cancer continues to steadily rise, proportionately with the HPV epidemic [1,21,22,23,24,25]. At time of diagnosis, most cases of anal cancer are at a localised stage with only 5–8% having distant metastases [15,26]. There are high curative rates associated with locoregional anal SCC treated with definitive chemoradiotherapy (dCRT), and survival rates have improved over recent years [15,27]. However, this is heavily influenced by patient-related factors including gender, socioeconomic status, HIV and human papillomavirus (HPV) status, and disease-related factors including tumour size, differentiation, and nodal involvement [11,28].

Nuances in these factors are difficult to clarify and a number of patients with anal cancer still require salvage abdomino-perineal resection (APR) following dCRT due to inadequate response to treatment or disease recurrence [29,30]. Thus, there is an emerging role for technology including radiomics and genomics to be used in combination with clinical risk factors to identify early indicators of treatment resistance or recurrence and tailor treatments in anal cancer [31,32].

The aim of this study is to ascertain factors associated with poorer survival in this Irish patient cohort and in doing so, improve survival outcomes for patients with anal cancer.

## 2. Methods

### 2.1. Study Design

A retrospective review of all patients being treated for anal cancer during the year 2016 to 2023, inclusive, was performed in an Irish hospital setting. We examined patient demographics, clinical presentation, disease characteristics, treatment modalities, and survival outcomes.

### 2.2. Data Collection and Ethical Approval

Medical records of all patients diagnosed with anal cancer during the specified seven-year period were identified and reviewed. Data were collected using a structured data collection form specifically designed for this study. Patient information was de-identified and anonymised to ensure privacy and compliance with ethical guidelines. Ethical approval was granted for this study by SJH/TUH Joint Research Ethics Committee, the Institutional Review Board (IRB) at this centre. Patient data were anonymised and maintained confidentially to ensure privacy and comply with relevant data protection regulations.

### 2.3. Inclusion Criteria

Patients who met the following criteria were included in this review:

1. Confirmed diagnosis of anal cancer (primary or recurrence).
2. Diagnosis between 1 January 2016 and 31 December 2023.
3. Availability of complete medical records, including demographic information, clinical notes, imaging reports, and treatment details.

### 2.4. Outcomes

The following data variables were collected for each patient:

- Primary outcome

Overall survival (OS) in days censored at last known follow up or date of known death.

- Secondary outcomes
  I. Demographic Information including sex, body mass index (BMI), HIV status, and age at diagnosis
  II. Clinical Presentation and Histopathological Subtype
  III. Treatment Modalities
  IV. Determination of any possible statistically significant predictors of censored survival using regression analysis.

### 2.5. Statistical Analysis

Descriptive statistics, such as means, medians, frequencies, and percentages, were used to summarise patient demographics, clinical presentation, and treatment modalities. Variables are reported as medians and ranges or means and standard deviations. Where dichotomous data are present, these values are reported as a number and a percentage of the total. The number of deaths observed is also reported. Mortality was treated as a time to event response, with data censored at last known follow up or date of known death. Missing values were not imputed. Kaplan–Meier survival curves are reported for covariates which showed statistically significant differences in survival on log-rank testing. Univariate and multivariable Cox proportional hazards models were used for the time to event outcome, taken as mortality or last date of follow up [33]. Hazard ratios were adjusted using covariates deemed clinically significant. Covariates with a low number of events or that made the statistical model unstable were excluded. Multivariable and univariate Cox regression hazard ratios are reported along with confidence intervals and *p*-values.

## 3. Results

### 3.1. Demographic Characteristics

In total, 75 patients were treated for anal cancer between 2016 and 2023. The median follow-up time for the study was 29.8 months. The median age at diagnosis was 63.4 years (range: 36–94). Female sex was more prevalent (57.3% (43/75)). The median BMI was 27.2 (17.3–48). Overall, 10.7% (8/75) were HIV positive patients and 93.8% (45/48) had documented HPV infection on histopathology (Table 1).

### 3.2. Clinical Presentation and Histopathological Subtype

Overall, the most common form of referral was from the general practitioner (44% (33/75). Histopathological subtype was predominantly squamous cell carcinoma (SCC) (88% (66/75)). The majority of those presented having locoregional disease (82.7% (62/75)), with 44 patients (58.7%) being staged as node negative disease. Metastatic disease was present in five patients (6.7%) at diagnosis. At the time of diagnosis and subsequent multidisciplinary team (MDT) discussion regarding oncological management, 63 underwent dCRT, 5 received palliative radiation, 3 were palliated, 2 proceeded to APR from outset, and 2 were undergoing MDT assessment.

### 3.3. Definitive Chemoradiation

The majority of patients underwent *dCRT* (84% (63/75)), with 71.4% of (45/63) patients having a complete response to dCRT. Of the patients where the MDT decision was to proceed with dCRT, eight did not undergo radiation therapy (and had chemotherapy alone). Those with adenocarcinoma histology (9.3%, 7/75) received neoadjuvant chemoradiation as part of their treatment regimen. The side effects experienced by the 63 patients following dCRT treatment included vulvovaginal and perineal mucositis (7.9% (5/63)), pedal oedema, peripheral neuropathy (4.8% (2/63)), groin abscess, faecal incontinence, radiation dermatitis and/or skin desquamation (14.3% (9/63)), radiation proctitis (7.9% (5/63)), and urinary tract infections (11.11% (7/63)) (Table 1).

### 3.4. Abdominoperineal Resection (APR) Group

Overall, nine patients underwent APR. Two patients proceeded straight to APR, one for refractory tumour bleeding, and the other having active inflammatory bowel disease and not suitable for radiation treatment. The remaining seven patients underwent dCRT with subsequent salvage APR given the residual tumour. The median age was 51 years (range: 39–79). Six operations were open, while two were laparoscopic. All cases involved anorectal excision, with four *en-bloc* multi-visceral resections (two vaginectomies, one urethrectomy and one prostatectomy). All resections had clear margins (R0). Six patients required flap reconstruction, all using vertical rectus abdominis myocutaneous (VRAM) flaps. The median number of nodes collected was 10 (3–17), with none having positive histology. Median hospital stay was 25 days.

### 3.5. Recurrence

The total recurrence rate post-dCRT was 17.46% (11/63). Local recurrence/re-growth was observed in six patients (8%), and distal recurrence was observed in five patients (6.7%). The sites of metastatic deposits were lung (n = 3, 4%), liver (n = 2, 2.7%), and sacral bone involvement (n = 1, 1.3%). Of the 11 patients with recurrence, 7 patients went on to have a salvage APR, with the remaining 4 patients receiving palliative care. Overall, five patients who had recurrence post-dCRT were alive at the time of manuscript preparation (up to 20 April 24). Those who had recurrence (5/11) had a worse survival compared to those who did not recur (10/64) (* *p* = 0.006) (Table 2).

### 3.6. Survival Influences

**Figure 1:**
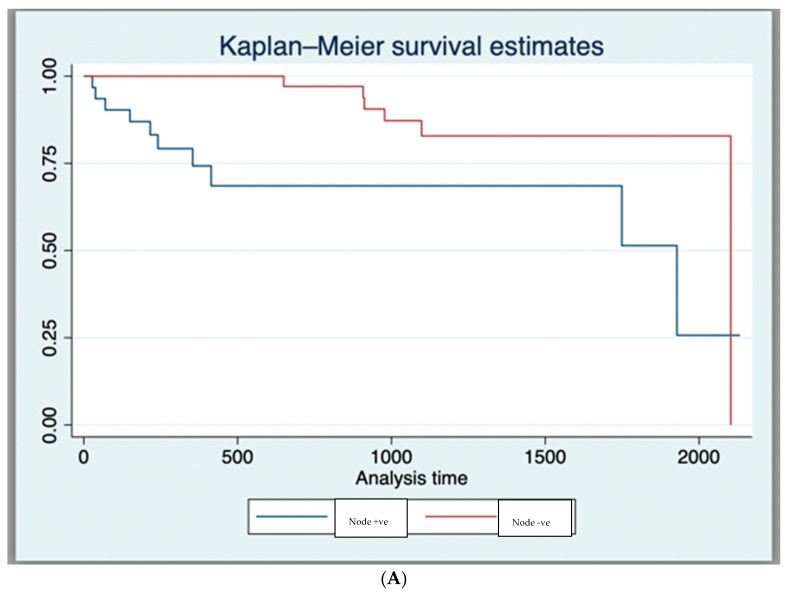

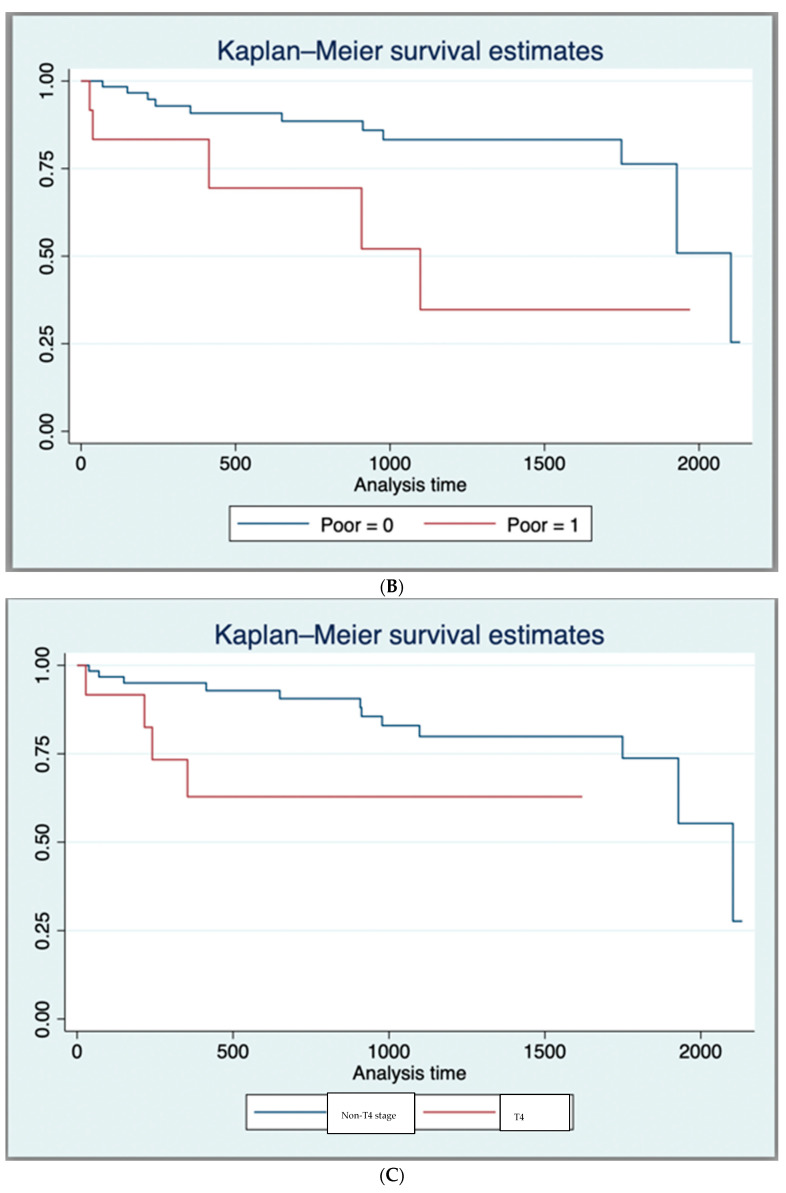

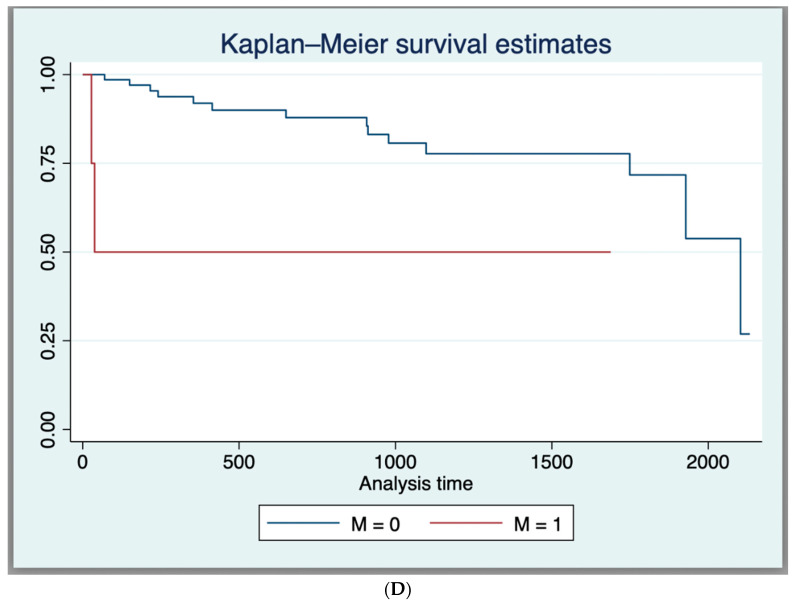
(**A**) Kaplan–Meier Survival Curves (Log-Rank Test Results (*p* < 0.05)) for nodal status. (**B**) Kaplan–Meier Survival Curves (Log-Rank Test Results (*p* < 0.05)) for poor histological differentiation. (**C**) Kaplan–Meier Survival Curves (Log-Rank Test Results (*p* < 0.05)) for T4 tumour stage. (**D**) Kaplan–Meier Survival Curves (Log-Rank Test Results (*p* < 0.05)) for metastasis at diagnosis.

The 1-year and 3-year OS for the entire cohort, as derived from the Kaplan–Meier curve, was 89.7% and 76.1%, respectively (Supplementary Material S2). In those having dCRT with complete response, survival was 93.2% (41/44), while for those requiring a salvage APR, survival was 57.14% (4/7). Expectantly, T4 tumours had a worse survival compared to those with other stages (* *p* = 0.02), as did those with nodal disease (* *p* = 0.02), metastases (* *p* = 0.001), or poor differentiation at the time of diagnosis (* *p* = 0.002) (Table 2 and Figure 1A–D). 

The following factors were associated with better survival on univariate analysis: complete response to dCRT HR = 0.058, (95% CI 0.012–0.28, * *p* = 0.0001), negative nodal status HR = 0.29, (95% CI 1 0.11–0.82, * *p* = 0.02). The following factors were associated with poorer survival on univariate analysis: T4 disease HR = 3.81, (95% CI 1.13–12.83, * *p* = 0.04), poorly differentiated tumour disease HR = 3.37, (95% CI 1.13–10.02, * *p* = 0.04), having positive nodal status HR = 5.03, (95% CI 1.11–22.8, * *p* = 0.04), and having metastatic disease at diagnosis HR = 5.8, (95% CI 1.28–26.42, * *p* = 0.02) (Table 2).

In the multivariable analysis, we examined the impact of several variables on survival, including male sex, tumour stage, nodal status, complete response to dCRT, recurrence, salvage APR, and poorly differentiated histology. The results indicated that, when controlled for all the co-variables listed above, complete response to dCRT (HR = 0.047, (95% CI: 0.004–0.522, * *p* = 0.013) and N0 status (HR = 0.0017, (95% CI: 0.00002–0.14, * *p* = 0.005) were found to be statistically significant positive predictors of survival. Male sex HR = 27.5, (95% CI: 2.53–298.78, * *p* = 0.006) was a statistically significant negative predictor of survival. (Supplementary Material S1).

## 4. Discussion

As seen in the literature, our retrospective review observed that stage, nodal, and differentiation status remain key prognostic indicators in those diagnosed with anal cancer [28,34,35,36,37]. Lu et al. conducted a multicentre study (11 cancer centres), involving anal cancer patient. Their univariable analysis showed that T stage significantly predicted recurrence-free survival (RFS) ([HR] = 3.03, 95% CI: 1.10–8.37, *p* = 0.032). Additionally, they found that N stage (HR = 3.05, 95% CI: 1.07–8.74, *p* = 0.038) was a significant predictor of OS [36]. Anal cancer is more common in elderly cohorts (median age = 63.4 years) and in females (57.3%), which is consistent with existing literature [38,39]. Expectantly, HIV positivity and HPV infection were prevalent in this cohort (in 10.7% and 93.8% of patients, respectively) [40,41,42].

The 3-year OS for the entire cohort was 76.1%, reducing to 57.14% in those requiring salvage APR. This is consistent with survival rates in other international centres [43,44,45].

The majority of patients (n = 63) presented with early disease (<T4), and 44 patients were deemed node negative. This likely reflects effective screening in those with high-risk features, and improved education programmes. A group of HIV care experts has issued the first U.S. federal guidelines designed to prevent anal cancer in people with HIV. These new recommendations advocate for a screening program that utilises high resolution anoscopy to identify and treat precancerous conditions, thereby preventing the development of anal cancer in individuals with HIV [46,47,48]. Expectantly, the predominance of SCC was the main histology of anal cancer, with >80% being well or moderately differentiated, consistent with other reported values [1,22,49].

Screening programmes for anal cancer are essential for early detection and improving survival outcomes, particularly among high-risk populations, working to effectively identify pre-cancerous lesions, such as anal intraepithelial neoplasia (AIN), before they progress to invasive cancer, alongside education and surveillance for high-risk groups [50,51,52]. There is a role for infectious disease clinics to screen high-risk cohorts, including individuals with HIV, HPV, vulvar intraepithelial neoplasia (VIN), and cervical intraepithelial neoplasia (CIN) [47,52]. Our study's findings align with previous research highlighting the importance of early detection in improving prognosis, particularly in patients with localised disease. In the cohort study by Leclerc et al., 700 HIV-positive patients were included. Out of these, 336 patients had at least one proctology visit. Anal cancer was diagnosed in 13 patients. Notably, among the patients who strictly adhered to the screening programmes (4.6%), no cases of AIN or anal cancer were reported [53].

HPV vaccination is critical for HIV-positive individuals and men who have sex with men (MSM), as both groups have a heightened risk of HPV-related anal cancer [41,54]. In HIV-positive individuals, the vaccine significantly reduces the prevalence of persistent HPV infections and associated precancerous lesions, thereby lowering cancer risk [46,54]. Similarly, research indicates that among MSM, HPV vaccination decreases the prevalence of high-risk HPV strains and the incidence of anal intraepithelial neoplasia (AIN), a precursor to anal cancer [55].

dCRT remains the cornerstone of anal cancer treatment, with 84% of patients in this cohort undergoing dCRT, in keeping with international standards [56]. Overall, 68.3% had dCRT alone, with 28.57% (18/63) having an incomplete response and 17.46% (11/63) developing a recurrence. Of these, 11.11% (7/63) underwent salvage APR. The multivariable analysis highlighted key significant predictors of survival. Complete response to dCRT, advanced disease stage at diagnosis (T4), poorly differentiation of the neoplasm, nodal involvement (N2), and metastatic disease were significant factors influencing outcomes. These findings are consistent with other international published results [28,34,35,36,37,57].

Recurrence remains a significant challenge in the management of anal cancer, with 8% of patients experiencing local recurrence and 6.7% developing distant metastases. Our analysis showed that recurrence was a strong predictor of poor survival (*p* = 0.006), emphasising the need for vigilant post-treatment surveillance and potential adjuvant therapies to mitigate the risk of recurrence. Interestingly, despite the poor prognosis generally associated with recurrent disease, five out of eleven patients with recurrence were still alive at follow-up, suggesting that aggressive management and salvage treatments can provide meaningful survival benefits in select patients.

The use of advanced adjuvants in monitoring the response to dCRT for anal cancer presents promising avenues for improving patient outcomes. Circulating tumour DNA (ctDNA) as a biomarker has shown potential in providing real-time insights into treatment efficacy and detecting minimal residual disease [58,59,60,61]. Our study underscores the importance of comprehensive monitoring, as 11.1% of patients required salvage APR for residual or recurrent disease, highlighting the need for more precise surveillance methods.

Radiomics is another noteworthy advancement in the role of prediction of treatment responses in anal cancer [62,63,64,65]. We have previously shown in a systematic review that radiomics-based risk stratification models were found to provide valuable insights into treatment response and patient outcomes, with all developed signatures demonstrating at least modest accuracy (range AUC: 0.68–1.0) in predicting their primary outcome in anal cancer [32]. The integration of these technologies into routine clinical practice could refine treatment plans and potentially reduce the need for invasive procedures, thereby improving OS rates and quality of life for patients with anal cancer.

We acknowledge that our study has some limitations, especially relating to sample size. This is not unexpected given the relative rarity of this disease. However, the longitudinal follow-up of this cohort has helped identify several critical factors influencing survival. Some findings may be clinically significant even though they are presented as not statistically significant above. These findings reinforce the importance of early detection, precise staging, and aggressive management of residual or recurrent disease following dCRT. Future research should focus on refining therapeutic strategies and optimising surveillance especially in high-risk groups.

## 5. Conclusions

This study highlights the critical prognostic factors in anal cancer management, including tumour stage, nodal status, and histological differentiation. Continued research into predictive biomarkers and advanced therapeutic approaches is essential to stratifying at-risk patients and optimising care.

## Figures and Tables

**Table 1:** Patient characteristics and demographics.

**Gender**	Male	32 (42.7%)
	Female	43 (57.3%)
**Age at diagnosis (years)**	Median	63
	Range	(36–94)
**T-stage**	Non-T4 stage	62 (82.7%)
	T4 stage	13 (17.3%)
**N-stage**	Negative	44 (58.7%)
	Positive	31 (41.3%)
**M-stage**	No metastasis	70 (93.3%)
	Metastasis	5 (6.7%)
**BMI Mean and SD**	*Mean and SD*	27.4 +/− 6.9
**Pathology**	SCC	66 (88%)
	AC	7 (9.3%)
	In-situ	1 (1.3%)
	HSIL	1 (1.3%)
**Differentiation**	Poor	12 (16%)
	Moderate	51 (68%)
	Well	12 (16%)
**Infectious disease**	HIV positive	8 (10.7%)
	HPV positive	45/48 (93.8%)
**Defunctioning stoma for dCRT**	Yes	22 (29.3%)
	No	53 (70.7%)

T = tumour size, M = metastases, N = nodal status, SCC = squamous cell carcinoma, AC = adenocarcinoma, HSIL = high-grade squamous intraepithelial lesions, BMI: body mass index, SD; standard deviation, HIV; human immunodeficiency virus, HPV; human papillomavirus, dCRT; definitive chemoradiotherapy.

**Table 2:** Univariate Analysis of Variables Associated with Survival and Log-Rank Test *p*-values.

		Number and %	Deaths	Significant *p* (log Rank)	HR (Univariate)	LCI	UCI	*p* Value
**Male**	No	43 (57))	6					
	Yes	32 (43)	9	0.162	2.03	0.17	5.6	0.16
**Age < 50**	No	64 (85)	13					
	Yes	11 (15)	3	* 0.03	0.89	0.24	3.25	0.86
**Non-T4 stage**	No	52 (69)	14					
	Yes	23 (31)	2	* 0.04	0.89	0.24	3.25	0.86
**T4**	No	63 (84)	12					
	Yes	12 (16)	4	* 0.02	3.81	1.13	12.83	* 0.04
**N**	No	44 (59)	6					
	Yes	31 (41)	10	* 0.02	5.03	1.11	22.8	* 0.04
**M**	No	71 (95)	14					
	Yes	4 (5)	2	* 0.001	2.32	0.849	6.37	0.11
**BMI < 25**	No	53 (71)	10					
	Yes	22 (29)	6	0.49	5.8	1.27	26.45	0.06
**Well differentiated**	No	63 (84)	15					
	Yes	12 (16)	1	0.23	0.307	0.04	2.36	0.18
**Mod differentiated**	No	24 (32)	5					
	Yes	51 (68)	9	0.54	0.73	0.26	2.04	0.55
**Poor differentiated**	No	63 (84)	11					
	Yes	12 (16)	5	* 0.02	3.37	1.13	10.02	* 0.04
**HIV**	No	67 (89)	13					
	Yes	8 (11)	3	0.54	0.61	0.12	3.11	0.53
**Defunctioning stoma for dCRT**	No	53 (71)	11					
	Yes	22 (29)	5	0.46	1.47	0.52	4.12	0.47
**Stoma reversal**	No	74 (99)	16					
	Yes	1 (1)	0	0.54	/	/	/	/
**dCRT**	No	12 (16)	5					
	Yes	63 (84)	11	0.23	0.47	0.13	1.78	0.28
**Palliative**	No	70 (93)	14					
	Yes	5 (7)	2	/	/	/	/	
**Salvage APR**	No	69 (92)	14					
	Yes	6 (8)	2	* 0.01	2.82	0.77	10.35	0.15
**Complete response to dCRT**	No	20 (32)	6					
	Yes	43 (68)	3	* 0.00001	0.058	0.012	0.28	* 0.0001

HR: Hazard Ratio, LCI: Lower Confidence Interval, UCI: Upper Confidence Interval, *p*: Probability value, BMI: Body Mass Index, HIV: Human Immunodeficiency Virus, dCRT: Definitive Chemoradiotherapy, APR: Abdominoperineal Resection. * are for all the p values that are less than 0.05.

## Data Availability

No new data were created or analysed in this study.

## Supplementary Materials

The following supporting information can be downloaded at: https://www.mdpi.com/article/10.3390/curroncol31090381/s1, Supplementary Material S1: Multivariable Analysis of Factors Affecting Survival. Supplementary Material S2: Kaplan-Meier survival estimates.
