# Supplementary material for: Factors Influencing Outcomes and Survival in Anal Cancer
Source: Curr Oncol. 2024 Sep 2;31(9):5151–63. doi: 10.3390/curroncol31090381 (PMC11431442; doi:10.3390/curroncol31090381)
Supplement: Supplementary file 1 [file curroncol-31-00381-s001.zip › curroncol-3142516-supplementary.pdf]

Supplementary Material

```
No. of subjects =      65                           Number of obs =      65
No. of failures =      13
Time at risk    = 62,434
                                                    LR chi2(10)   =   36.80
Log likelihood = -23.957164                         Prob > chi2   =  0.0001
```

_t	Haz. ratio	Std. err.	z	P>|z|	[95% conf.	interval]
Sex	27.51044	33.47845	2.72	0.006	2.533025	298.7828
T1	.5861841	1.176965	-0.27	0.790	.011454	29.99938
T2	.8733099	1.052158	-0.11	0.910	.0823468	9.261688
T3	.2748562	.3680955	-0.96	0.335	.0199137	3.793671
N0	.0017512	.0039615	-2.81	0.005	.0000208	.1475342
N1	.0591105	.0795513	-2.10	0.036	.0042278	.8264518
CompleteReponsedCRT	.0473446	.0579878	-2.49	0.013	.0042925	.5221859
Recurrence	1.419252	1.301733	0.38	0.703	.2351476	8.566013
dCRTthenAPR	7.880716	10.42204	1.56	0.119	.5900294	105.2586
Poor	.7965123	.8363727	-0.22	0.828	.101717	6.237223

Supplementary Material S1: Multivariable Analysis of Factors Affecting Survival

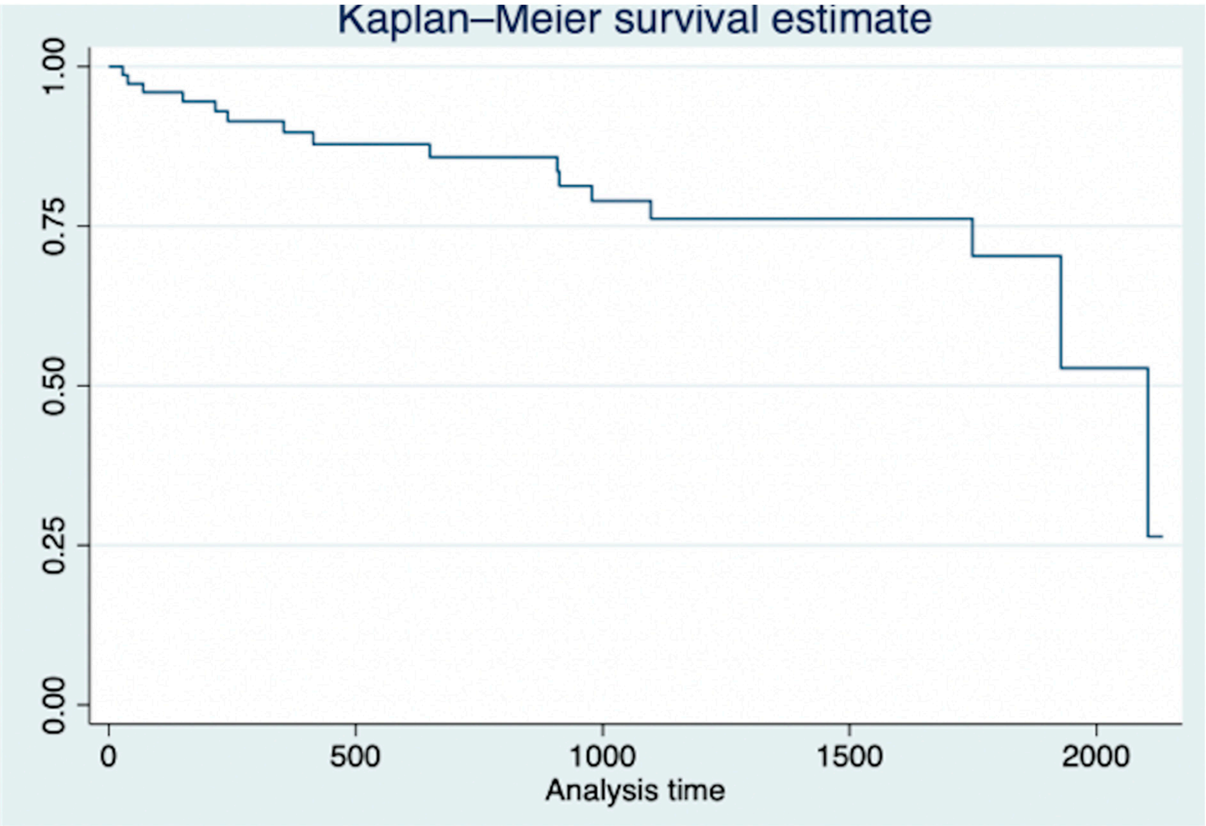

Supplementary Material S2: Kaplan-Meier survival estimates
